# The LEAP Program: Quality Improvement Training to Address Team Readiness Gaps Identified by Implementation Science Findings

**DOI:** 10.1007/s11606-020-06133-1

**Published:** 2020-09-08

**Authors:** Laura J. Damschroder, Nicholas R. Yankey, Claire H. Robinson, Michelle B. Freitag, Jennifer A. Burns, Susan D. Raffa, Julie C. Lowery

**Affiliations:** 1 Center for Clinical Management Research, VA Ann Arbor Healthcare System, Ann Arbor, MI USA; 2 National Center for Health Promotion and Disease Prevention, Veterans Health Administration, Washington, DC USA; 3 Department of Psychiatry & Behavioral Sciences, Duke University School of Medicine, Durham, NC USA

**Keywords:** quality improvement, implementation science, learning collaborative

## Abstract

**Background:**

Integrating evidence-based innovations (EBIs) into sustained use is challenging; most implementations in health systems fail. Increasing frontline teams’ quality improvement (QI) capability may increase the implementation readiness and success of EBI implementation.

**Objectives:**

Develop a QI training program (“Learn. Engage. Act. Process.” (LEAP)) and evaluate its impact on frontline obesity treatment teams to improve treatment delivered within the Veterans Health Administration (VHA).

**Design:**

This was a pre-post evaluation of the LEAP program. MOVE! coordinators (*N* = 68) were invited to participate in LEAP; 24 were randomly assigned to four starting times. MOVE! coordinators formed teams to work on improvement aims. Pre-post surveys assessed team organizational readiness for implementing change and self-rated QI skills. Program satisfaction, assignment completion, and aim achievement were also evaluated.

**Participants:**

VHA facility-based MOVE! teams.

**Interventions:**

LEAP is a 21-week QI training program. Core components include audit and feedback reports, structured curriculum, coaching and learning community, and online platform.

**Main Measures:**

Organizational readiness for implementing change (ORIC); self-rated QI skills before and after LEAP; assignment completion and aim achievement; program satisfaction.

**Key Results:**

Seventeen of 24 randomized teams participated in LEAP. Participants' self-ratings across six categories of QI skills increased after completing LEAP (*p*< 0.0001). The ORIC measure showed no statistically significant change overall; the change efficacy subscale marginally improved (*p* < 0.08), and the change commitment subscale remained the same (*p* = 0.66). Depending on the assignment, 35 to 100% of teams completed the assignment. Nine teams achieved their aim. Most team members were satisfied or very satisfied (81–89%) with the LEAP components, 74% intended to continue using QI methods, and 81% planned to continue improvement work.

**Conclusions:**

LEAP is scalable and does not require travel or time away from clinical responsibilities. While QI skills improved among participating teams and most completed the work, they struggled to do so amid competing clinical priorities.

**Electronic supplementary material:**

The online version of this article (10.1007/s11606-020-06133-1) contains supplementary material, which is available to authorized users.

## INTRODUCTION

Integrating evidence-based innovations (EBIs) into sustained use is a well-documented challenge for health systems. Organizational leaders report that most efforts to implement change fail.^1, 2^ Implementation science (IS) emphasizes closing the gap between evidence and practice,^3^ usually within a prescribed period of time.^4^ For example, obesity and related lifestyle behaviors like poor diet and physical inactivity impact life expectancy. Among US veterans, the prevalence of obesity has been documented at 41%, higher than that of the US population and higher than that of the previous decade. Despite strong evidence that comprehensive lifestyle interventions (CLIs) are effective treatment for obesity, these interventions are not reliably delivered to patients who would benefit.

The Veterans Health Administration (VHA) is one of the largest integrated health systems in the world, serving about six million enrolled veterans.^5^ In 2006, VA established the MOVE! Weight Management Program for Veterans (MOVE!), aligned with the strong evidence base for CLIs.^6, 7^ MOVE! has demonstrated modest short-term weight loss.^8, 9^ Though coordinators who lead local MOVE! programs at facilities across VHA have access to the robust CLI and MOVE! evidence base, plus implementation resources,^10^ program delivery is highly variable across VHA.^11, 12^ While local clinicians have worked diligently to implement MOVE! at their respective facilities, much work remains to optimize program delivery to improve patient outcomes.

We have published four implementation evaluations of four CLIs, including MOVE!.^12–15^ Each evaluation was guided by the Consolidated Framework for Implementation Research (CFIR),^16^ which describes contextual factors that arise as barriers to successful program implementation. Our evaluations consistently revealed barriers related to lack of the following: (1) planning, (2) engaging key individuals, and (3) reflecting and evaluating on progress and impact. The CFIR highlights that these activities “…can be accomplished in any order [using an] incremental approach to implementation; e.g., *using a plan-do-study-act* [PDSA] approach to incremental testing. [emphasis added].”^16^ These findings reinforce our contention that frontline staff should drive efforts to achieve optimal program implementation to benefit their patients.

**Figure 1:**
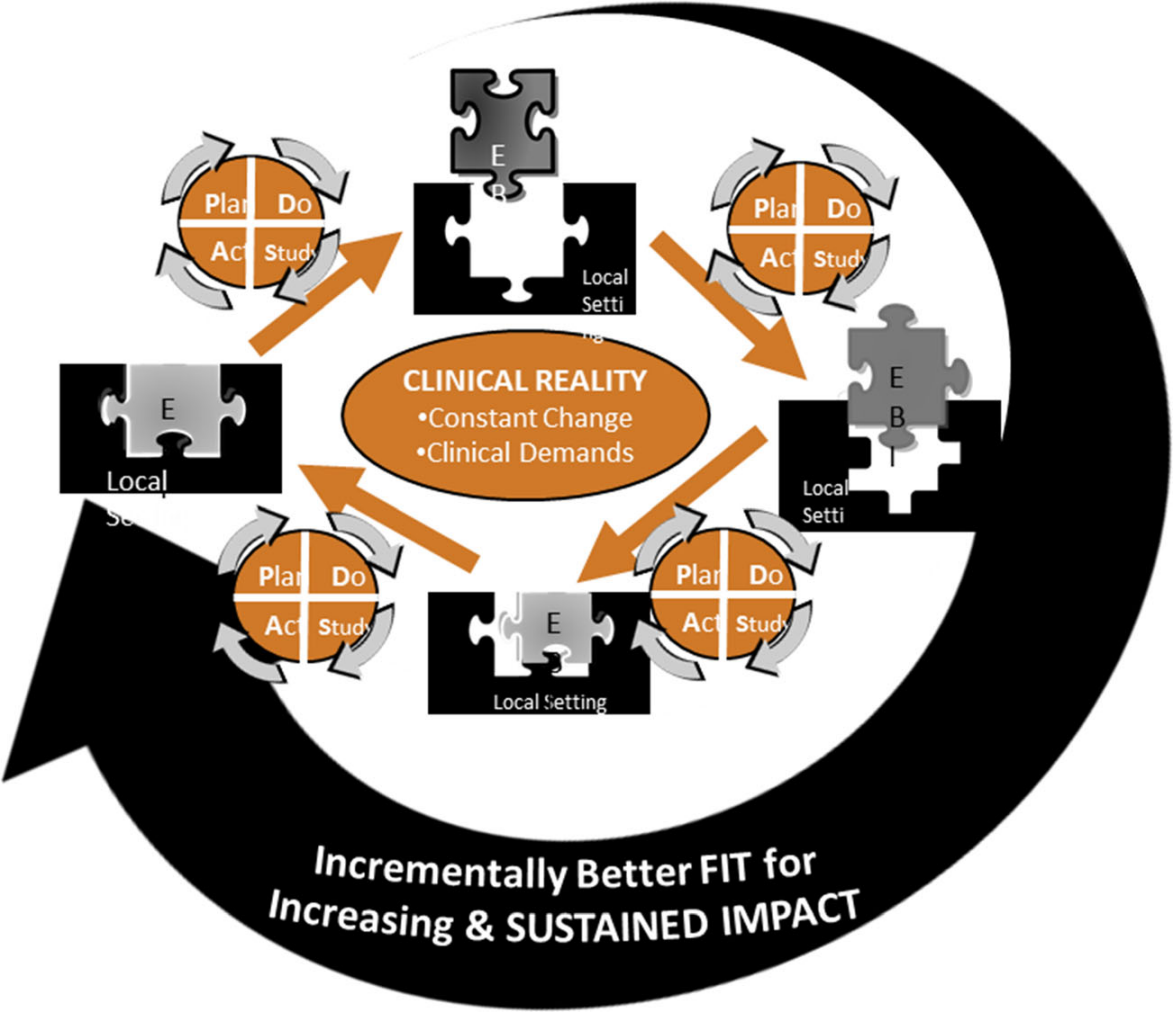
**Dynamic Sustainability Framework (DSF).**

The Dynamic Sustainability Framework (DSF)^17^ is a helpful conceptual framework for guiding optimized implementations to overcome identified barriers (Fig. 1). The DSF asserts that organizational learning should be a core value because of the need to continue to optimize the intervention over time. Thus, implementation cannot be just a one-time burst of effort but rather a dynamic process that engages teams over time. It places PDSA cycles of change at the heart of sustained implementation and reinforces that PDSAs are critical for accomplishing planning, engaging key individuals, and reflecting and evaluating progress and impact. With its focus on PDSAs, the DSF points squarely to the use of quality improvement (QI) techniques as a means of sustaining CLI implementation. Through PDSA cycles, frontline providers can systematically identify and address the barriers that prevent MOVE! from being optimally delivered. Based on the DSF, fully optimized implementation of MOVE! will lead to better patient outcomes.^6, 7^ Our goal was not to change the fundamental content and structure of MOVE! but rather to address variability in program delivery because, based on our earlier work, it was apparent that clinicians struggled to optimize their program to adapt to local needs and align with clinical practice guidelines for weight management.

Drawing on the DSF, the objective of this project was to train frontline clinical teams in QI to dynamically optimize MOVE! delivery. In busy clinical work environments, MOVE! clinicians need a QI training program that meets the following criteria: (1) provides easy-to-understand and accessible content, (2) allows hands-on learning within a busy clinical setting, and (3) provides coaching support and a supporting learning community to enhance learning and accountability. The specific aim of this study was to evaluate the “Learn. Engage. Act. Process.” (LEAP) program, which was developed to address these requirements.

## METHODS

This is a pre-post study to assess the capability of teams to conduct PDSA cycles and improve readiness for implementation. This study was deemed a non-research operations activity and follows the Standards for Reporting Implementation Studies (StaRI).^18^

### Team Recruitment

MOVE! program sites were selected via random selection, stratified by patient participation rate (calculated as the number of patients who completed at least one group-based MOVE! session in the first six months of FY 2016 divided by the number of patients eligible for MOVE!). MOVE! programs were categorized as high reach (above median participation rate) versus low reach (below median participation rate). Half of MOVE! programs were randomly selected to receive an e-mail invitation to participate in LEAP in fiscal year 2017. Twenty-four programs were randomly selected from the list of MOVE! coordinators who expressed interest in participating in LEAP and assigned to one of four starting dates.

### LEAP Intervention

LEAP is a virtual, structured training program that trains frontline teams how to run PDSAs through coaching with hands-on learning as teams develop and execute a Project Charter (see Appendix 1) to complete a PDSA cycle of change. Teams were assigned to cohorts with five teams from other MOVE! programs within VHA, to establish a learning community. The LEAP curriculum was adapted for teams and streamlined for busy clinical settings based on a Massive-Open Online Course (MOOC) developed by HarvardX in collaboration with IHI.^19^ Appendix 2 provides a brief summary of LEAP’s curriculum.

LEAP spanned 21 weeks to give teams the time to learn and apply new skills while simultaneously executing an initial cycle of change. LEAP includes hour-long coaching calls or virtual collaboratives (VCs) involving teams with MOVE! programs from other facilities each week, to enhance learning and accountability. Teams are encouraged to convene local weekly meetings to learn and apply the curriculum to their own project; teams complete assignments, review data, and plan for future PDSAs. Teams did not have formally allocated time to dedicate to LEAP or QI activities. Each individual, however, committed to carving out 2–4 h per week for LEAP. At the end of LEAP, all teams gave a final presentation in a VC and received feedback from their coaches and peer teams within the collaborative. The final presentations included plans for their next PDSA cycle; each team was given a completion certificate and a copy of *The Improvement Guide*.^20^

Data-driven PDSAs require program metrics to help teams identify improvement opportunities. A user-centered design approach guided development of audit and feedback reports for MOVE! teams. Personas^21^ were developed based on semi-structured interviews with MOVE! program leaders to capture their data and reporting needs. The study team designed reports based on this input and refreshed them monthly or quarterly.

### Measures

#### Organizational Readiness for Implementing Change

Organizational readiness for implementing change (ORIC) refers to team members’ shared determination to implement a change (change commitment) and shared belief in their collective capability to do so (change efficacy).^22^ Shea and colleagues developed and validated a 12-item instrument with two scales: (1) collective change efficacy (seven items, Cronbach’s reliability *ɑ* = 0.93) and (2) collective change commitment (five items, Cronbach’s reliability *ɑ* = 0.95). A 5-point Likert scale ranging from disagree to agree (1 to 5 points) was used to assess each item (see Appendix 3).

#### Self-Rated Quality Improvement Skills

A self-assessment, adapted from *The Improvement Guide*^20^ and the IHI Improvement Coach Professional Development program,^23^ was used to elicit participant ratings of confidence in applying QI methods using a six-point scale ranging from “no knowledge” to “expert” (see Appendix 4). The survey consisted of 19 items across the following topics: (1) support a change with data (4 items), (2) develop a change (4 items), (3) test a change (3 items), (4) implement a change (2 items), (5) spread a change (2 items), and (6) the human side of change (4 items).

#### Assignment Completion and Aim Achievement

Assignment completion was tracked based on materials uploaded by each team to the online platform. Aim achievement was determined based on each team’s final presentation of progress toward achieving their aim. Teams were encouraged to collect data for at least 12 time points to assess reliability.

#### Program Satisfaction

Upon completion of LEAP, a 21-item survey of satisfaction across five LEAP domains (improvement coach support, quality of curriculum materials, organization of materials online, number of assignments, and technology requirements of the program) and future intentions to continue with QI was administered to team members (see Appendix 5). Comment space was available for additional feedback. Responses were summarized for each cohort and shared in the final week’s VC.

#### Data Collection

Surveys were administered online via Qualtrics survey software (Qualtrics, LLC, Provo, UT). Surveys of organizational readiness and QI skills were completed in weeks 4 and 6 of LEAP, respectively, and again at the end of LEAP. The satisfaction survey was administered in week 20.

#### Data Analysis

Descriptive statistics were generated for all measures. Paired *t* tests were used to test for differences in readiness and QI skill ratings from early in the program to completion of LEAP. Analyses were conducted using SAS 9.4 (SAS Institute, Inc., Cary, NC).

## RESULTS

### Participation

**Figure 2:**
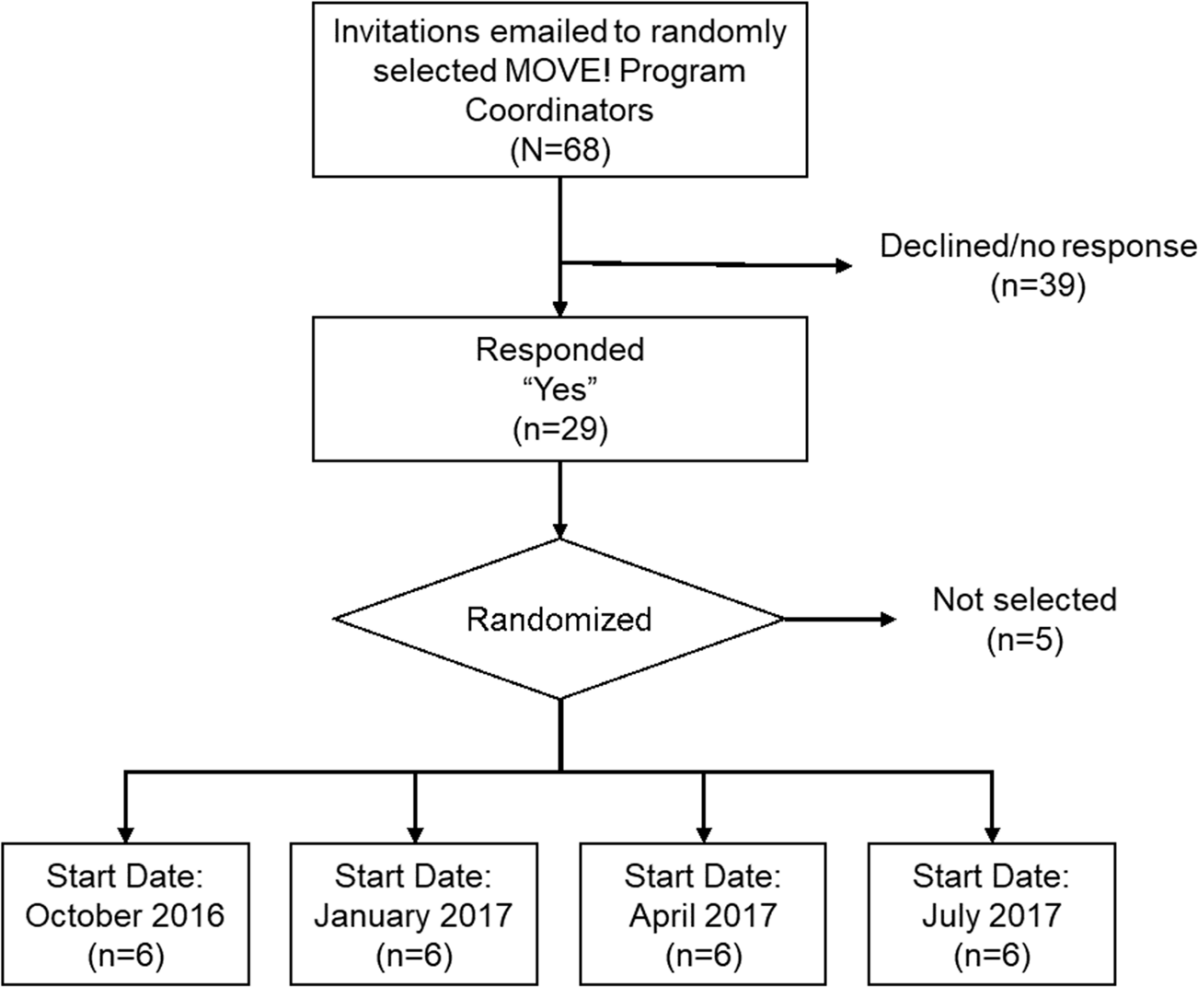
**MOVE! Program team recruitment flow.**

Invitations were emailed to MOVE! coordinators of 68 randomly selected group MOVE! programs. As shown in Figure 2, 29 coordinators (43%) responded “yes” and 24 coordinators were randomly chosen and assigned to one of the four start dates; of these, 71% (*n* = 17) participated. Table 1 characterizes the 17 MOVE! programs involved. Contrary to evidence-based policy guidance, 59% (10/17 teams) reported open (instead of closed) enrollment, and 30% (5/17 teams) offered fewer than the recommended 16 sessions in their standard curriculum.

**Table 1:** Characteristics of Participating Programs and Teams (*n* = 17)

Characteristics	*n* (%)
MOVE! program coordinator is a dietitian	16 (94)
Program has open enrollment*	10 (59)
Number of sessions^†^	
16	12 (70)
12	3 (18)
< 12	2 (12)

*Open enrollment means a participant may join a group at any time

^†^The number of sessions offered

LEAP coaches worked with the team leader (the MOVE! coordinator was nearly always the team leader for LEAP) to build their team by inviting colleagues. Altogether, 97 individuals participated across the 17 teams, an average of 5.8 (1–9) individuals per team. Most LEAP team members were dietitians or nurses (see Table 2).

**Table 2:** Participant Profession

Profession	*n*
Dietitian	45
Nurse	16
Physical/occupational/recreational therapist	4
Psychologist	6
Patient	2
Other	16
Unknown	8
Total	97

### Self-Rated Quality Improvement Skills

Fifty-five participants, including all team leaders, completed the baseline self-rated QI skills assessment; 40 participants completed the follow-up assessment. Table 3 shows scores for baseline and follow-up for the 28 participants who completed the survey at both time points. Each of the measures increased significantly from early participation in LEAP to its completion. There were no significant differences in responses from participants who only responded at baseline (*n* = 27) versus those who completed both time points (*n* = 28).

**Table 3:** Organizational Readiness for Implementing Change (ORIC) and Self-Rated QI Skill Assessment

Scale	Baseline	Follow-up	Difference	*p* value
Organizational readiness for implementing change (*n* = 23)				
Change commitment	4.22 (0.62)	4.29 (0.67)	0.07	0.6603
Change efficacy	3.93 (0.73)	4.18 (0.47)	0.25	0.0782
Self-rated QI skill assessment (*n* = 28)				
Support a change with data	2.47 (1.17)	3.82 (0.56)	1.35	< 0.0001
Develop a change	2.94 (1.14)	3.94 (0.69)	1.00	< 0.0001
Test a change	2.88 (1.25)	3.94 (0.71)	1.06	< 0.0001
Implementing a change	2.95 (1.18)	3.93 (0.87)	0.98	< 0.0001
Spreading a change	2.80 (1.29)	3.93 (0.99)	1.13	< 0.0001
Human side of change	3.28 (1.04)	4.04 (0.69)	0.76	< 0.0001

### Organizational Readiness for Implementing Change

There was a marginally significant increase in change efficacy (*p* < 0.08) but not change commitment (*p* = 0.66) (see Table 3). There were no significant baseline differences for individuals who only responded at baseline (*n* = 16) versus those who completed both time points (*n* = 23).

### Assignment Completion and Aim Achievement

Assignment completion varied widely from 35 to 100% (Table 4). For example, all teams uploaded their project charter, but only 35% uploaded a process map. Completion rates may be underestimated because teams completed assignments but did not upload them to the online platform.

**Table 4:** Completion of LEAP Assignments

Assignment	Completion: *n* (%)
Team member roles	9 (53)
Affinity diagram	10 (58)
Matrix diagram	11 (65)
Fishbone diagram	12 (71)
First half of charter	16 (94)
Process map	6 (35)
Project charter	17 (100)
Project charter feedback for another team	11 (65)
Run chart	14 (82)
Final presentation	16 (94)

Teams developed improvement aims related to eight topics; most focused on increasing MOVE! enrollments (*n* = 11). Teams also worked to improve patient retention, decrease wait times, improve documentation of weights, improve weight outcomes, increase patient goal setting, or increase patients’ use of logs to record physical activity. Nine teams reported they had achieved their aim, though some of those teams did not document enough data points to confirm.

### LEAP Program Satisfaction

Altogether, 55 out of 97 (57%) LEAP team members completed the satisfaction survey. Most participants were satisfied or very satisfied (81–89%) with all LEAP components. Comments described technical issues with navigating the online platform and unreliability and unavailability of webcams as common issues.

Though almost all respondents (96%) agreed or strongly agreed that LEAP was relevant to the needs of their MOVE! program, 53% disagreed that they had enough time to do the required work. Respondents commented that finding time and available staff to participate on the team were significant challenges.

Despite the reported lack of time, most respondents agreed or strongly agreed that they would have time to continue to apply LEAP methods to their MOVE! programs in the future (74%), their LEAP team would continue working together after completing LEAP (81%), and they planned to participate in future monthly sessions (57%). This was reinforced by comments that expressed a strong desire for continued availability of LEAP coaches, data reports, and help interpreting data to inform future improvements.

## DISCUSSION

This is a story about how implementation researchers, guided by the DSF, landed in the sphere of quality improvement in their quest to increase team readiness to optimize an evidence-based CLI within challenging clinical settings. We began by drawing on IS findings informed by the CFIR that pointed to key barriers. The CFIR and DSF frameworks pointed to a strategy of engaging teams in PDSAs as a pathway to optimize CLIs. This led to the design of the LEAP QI training program for frontline teams. Thus, LEAP is rooted within the intersection of IS and QI. LEAP draws on QI curriculum adapted for hands-on learning by frontline teams. Weight management is a function of both individual behavior change by patients and CLIs that are designed to help patients achieve outcome goals.^6^ LEAP increased capability of clinical teams to dynamically optimize MOVE! over time; the DSF asserts this is essential for continued program optimization.^17^

This study amplifies the challenge of integrating precepts of IS with QI. QI approaches are rooted in local expertise, leading to “bottom-up,” team-derived goals.^24^ In contrast, IS goals are often imposed “top-down” based on getting evidence into practice.^4^ The improvement aims teams selected often focused on increasing enrollments in reaction to disruptive system changes that dramatically impacted their referral processes. During this study, a “direct scheduling” approach was mandated so patients could enroll in MOVE! without a referral; enrollment workflows had to be redesigned to inform, invite, and assist patients. This was high priority for teams versus an IS goal of implementing/optimizing an evidence-based program component (e.g., closed versus open enrollment). Contributing processes like enrollment workflow are often overlooked in intervention design.^25, 26^ In fact, a recent implementation study involving over 1700 primary care practices recommended filling a void in a widely used compilation of implementation strategies^27, 28^ by adding workflow redesign to the list.^29^

It will take time to see measurable impacts on clinical outcomes (e.g., weight loss), which are commonly the focus of IS projects that are usually funded for a time period inadequate to capture slower but, in theory, better optimized and more sustainable implementations.^4, 17^ Not all LEAP teams met their aim, but even when teams did not achieve their aim, they communicated valuable lessons learned and felt better positioned to plan and execute future PDSAs; this is one of the main goals of PDSAs.^24^

The context in which LEAP was delivered is extremely important to consider. The MOVE! weight management program was not a high priority in VHA, relative to other higher priority issues, such as increasing access for patients,^30^ reducing suicide rates,^31^ addressing the opioid epidemic,^32^ and increasing access to community providers,^33^ all of which experienced focused media attention within and outside VHA during the course of this study. Regardless of topic, many organizations fail to sufficiently support investments in continuous QI by frontline clinicians/staff.^34–36^ Thus, a key design goal for LEAP was to coach frontline teams without requiring formal leadership commitment, leveraging a bottom-up “learn by doing” approach that could be accomplished within a demanding clinical setting and in the face of invisibility with mixed levels of support. Even with this approach, teams struggled with finding time to accomplish the work. Based on input from participating teams, we plan to extend the duration of LEAP to 26 weeks (without adding more content) to allow more time for teams to implement their planned change(s).

QI training is often delivered through intensive in-person, multi-day workshops or collaboratives requiring face-to-face interaction with other teams and coaches.^37–39^ Workshop-based training often fails to show impact because trainees do not apply this new knowledge within everyday clinical work.^40–43^ Virtual (internet- and/or phone-enabled) adaptations of collaboratives are increasingly used to mitigate participation barriers but often still require dedicated learning sessions and often last a year or more.^44–50^ One large-scale quality collaborative in VHA provided virtual monthly coaching but only after teams started executing their planned PDSA cycle.^47, 48^ LEAP coaches, in contrast, walked teams through every step, week by week, tailoring guidance to the needs of teams and providing feedback on interim progress. LEAP curriculum was paced; though team leaders might spend up to four hours per week on learning and activities, team members usually spent much less time.

Health system leaders are putting increasing attention and urgency on engaging frontline teams in QI to provide greater participation and control over clinical processes, and as a potential antidote for clinician burnout.^51, 52^ System leaders, however, continue to struggle to accomplish this^34–36, 53^ though there are pockets of success.^52, 54, 55^ More organizations are recognizing that investing in QI is “an enlightened strategy, not an expense;^52^^, p51^” LEAP can help engage teams in QI and increase readiness for implementing change in a constantly changing environment.^17^ We plan to partner with VHA Lean belt-trainers to understand circumstances under which Lean and LEAP produce team engagement in continuous QI. Continuous QI is increasingly pursued by healthcare systems^41, 43, 56–58^; it aligns with the assertions of the DSF to optimize interventions,^17^ and was a core strategy in one large-scale implementation initiative involving over 1700 primary care practices in the USA, but also encountered challenges.^29^

This study has several limitations. The goal of LEAP was to increase QI capability among teams leading the MOVE! weight management program. Developing this capability does not, however, address *capacity* to engage in QI, which requires a well-rooted learning culture and/or alignment with organizational priorities. LEAP did increase QI capability, but measurable impact on clinical outcomes will take time and will only happen if teams continue to engage in PDASs. Future work needs to focus on how to develop stronger alignment between frontline QI foci and organizational priorities to help resolve time constraints and the pressure from competing priorities experienced by teams.^41, 55, 59^

## CONCLUSION

LEAP was developed to address implementation gaps identified through IS-guided evaluations and embraces QI as a foundational strategy for frontline clinical teams to optimize an evidence-based intervention. LEAP is scalable and cost-saving (no travel required), does not require absence from clinical responsibilities, and increased teams’ QI *capability*. However, the *capacity* of teams to continue QI is challenged by nearly universal time constraints and competing clinical priorities.

## Electronic supplementary material

ESM 1 (DOCX 251 kb).

## References

[1] Meaney M, Pung C (2008). McKinsey global results: creating organizational transformations. McKinsey Q.

[2] Rafferty AE, Jimmieson NL, Armenakis AA (2013). Change readiness: a multilevel review. J Manag.

[3] Balas EA, Boren SA (2000). Managing clinical knowledge for health care improvement. Yearb Med Inform.

[4] Scheirer MA, Dearing JW (2011). An agenda for research on the sustainability of public health programs. Am J Public Health.

[5] VA Utilization Profile FY 2016. In: Affairs USDoV, ed: National Center for Veterans Analysis and Statistics; 2017.

[6] US Department of Veterans Affairs, US Department of Defense. VA/DoD clinical practice guideline for screening and management of overweight and obesity. Department of Veterans Affairs and Department of Defense; 2014.

[7] Patnode CD, Evans CV, Senger CA, Redmond N, Lin JS (2017). Behavioral counseling to promote a healthful diet and physical activity for cardiovascular disease prevention in adults without known cardiovascular disease risk factors: updated evidence report and systematic review for the US Preventive Services Task Force. Jama.

[8] Maciejewski ML, Shepherd-Banigan M, Raffa SD, Weidenbacher HJ (2018). Systematic review of behavioral weight management program MOVE! for veterans. Am J Prev Med.

[9] Kahwati LC, Lance TX, Jones KR, Kinsinger LS (2011). RE-AIM evaluation of the Veterans Health Administration's MOVE! weight management program. Transl Behav Med.

[10] US Department of Veterans Affairs. MOVE! weight management program. https://www.move.va.gov/. Published 2020. Accessed January 30, 2020.

[11] Maciejewski ML, Arterburn DE, Berkowitz TS (2019). Geographic variation in obesity, behavioral treatment, and bariatric surgery for veterans. Obesity.

[12] Damschroder LJ, Lowery JC (2013). Evaluation of a large-scale weight management program using the consolidated framework for implementation research (CFIR). Implement Sci.

[13] Damschroder LJ, Reardon CM, Sperber N, Robinson CH, Fickel JJ, Oddone EZ (2016). Implementation evaluation of the telephone lifestyle coaching (TLC) program: organizational factors associated with successful implementation. Transl Behav Med.

[14] Damschroder LJ, Reardon CM, AuYoung M (2017). Implementation findings from a hybrid III implementation-effectiveness trial of the Diabetes Prevention Program (DPP) in the Veterans Health Administration (VHA). Implement Sci.

[15] **Goodrich DE, Lowery JC, Burns JA, Richardson CR**. The phased implementation of a national telehealth weight management program for veterans: mixed-methods program evaluation. JMIR Diabetes*.* 2018;3(4). 10.2196/diabetes.9867 PMC6307696 30305265

[16] Damschroder LJ, Aron DC, Keith RE, Kirsh SR, Alexander JA, Lowery JC (2009). Fostering implementation of health services research findings into practice: a consolidated framework for advancing implementation science. Implement Sci.

[17] Chambers DA, Glasgow RE, Stange KC (2013). The dynamic sustainability framework: addressing the paradox of sustainment amid ongoing change. Implement Sci.

[18] **Pinnock H, Barwick M, Carpenter CR, et al.** Standards for Reporting Implementation Studies (StaRI) statement. BMJ*.* 2017;356. 10.1136/bmj.i6795 PMC5421438 28264797

[19] Practical improvement science in health care: a roadmap for getting results. EdX, Inc. https://www.edx.org/course/ph556x-practical-improvement-science-in-health-care-a-roadmap-for-getting-results. Accessed January 15, 2019.

[20] **Langley GJ, Moen RD, Nolan KM, Nolan TW, Norman CL, Provost LP**. The improvement guide: a practical approach to enhancing organizational performance*.* Wiley; 2009.

[21] **Parisi KE, Dopp AR, Munson SA, Lyon AR**. A glossary of user-centered design strategies for implementation experts. Transl Behav Med. 2019;9(6):1057-1064. 10.1093/tbm/iby119 30535343

[22] Shea CM, Jacobs SR, Esserman DA, Bruce K, Weiner BJ (2014). Organizational readiness for implementing change: a psychometric assessment of a new measure. Implement Sci.

[23] Institute for Healthcare Improvement (IHI). IHI Improvement Coach Professional Development Program. http://www.ihi.org/education/InPersonTraining/ImprovementCoach/Improvement-Coach/Pages/default.aspx. Published 2020. Accessed January 30, 2020.

[24] Perla RJ, Provost LP, Parry GJ (2013). Seven propositions of the science of improvement: exploring foundations. Qual Manag Health Care.

[25] Aziz Z, Absetz P, Oldroyd J, Pronk NP, Oldenburg B (2015). A systematic review of real-world diabetes prevention programs: learnings from the last 15 years. Implement Sci.

[26] Balk EM, Earley A, Raman G, Avendano EA, Pittas AG, Remington PL (2015). Combined diet and physical activity promotion programs to prevent type 2 diabetes among persons at increased risk: a systematic review for the Community Preventive Services Task Force. Ann Intern Med.

[27] Powell B, Waltz T, Chinman M (2015). A refined compilation of implementation strategies: results from the Expert Recommendations for Implementing Change (ERIC) project. Implement Sci.

[28] Waltz T, Powell B, Chinman M (2014). Expert recommendations for implementing change (ERIC): protocol for a mixed methods study. Implement Sci.

[29] Perry CK, Damschroder LJ, Hemler JR, Woodson TT, Ono SS, Cohen DJ (2019). Specifying and comparing implementation strategies across seven large implementation interventions: a practical application of theory. Implement Sci.

[30] Penn M, Bhatnagar S, Kuy S (2019). Comparison of wait times for new patients between the private sector and United States Department of Veterans Affairs Medical Centers. JAMA Netw Open.

[31] **Hoge CW**. Suicide reduction and research efforts in service members and veterans—sobering realities. JAMA Psychiat*.* 2019. 10.1001/jamapsychiatry.2018.4564 30758501

[32] Chinman M, Gellad WF, McCarthy S (2019). Protocol for evaluating the nationwide implementation of the VA Stratification Tool for Opioid Risk Management (STORM). Implement Sci.

[33] **Kesling B**. VA issues new rules expanding access to private care. Wall Street J*.* 2019.

[34] Vaughn VM, Saint S, Krein SL (2019). Characteristics of healthcare organisations struggling to improve quality: results from a systematic review of qualitative studies. BMJ Qual Saf.

[35] Swensen SJ, Dilling JA, Mc Carty PM, Bolton JW, Harper CM (2013). The business case for health-care quality improvement. J Patient Saf.

[36] Reed JE, Card AJ (2016). The problem with plan-do-study-act cycles. BMJ Qual Saf.

[37] Godfrey MM, Oliver BJ (2014). Accelerating the rate of improvement in cystic fibrosis care: contributions and insights of the learning and leadership collaborative. BMJ Qual Saf.

[38] von Benzon Hollesen R, Johansen RLR, Rørbye C, Munk L, Barker P, Kjaerbye-Thygesen A (2018). Successfully reducing newborn asphyxia in the labour unit in a large academic medical centre: a quality improvement project using statistical process control. BMJ Qual Saf.

[39] Glasgow JM, Davies ML, Kaboli PJ (2012). Findings from a national improvement collaborative: are improvements sustained?. BMJ Qual Saf.

[40] Simpson DD (2002). A conceptual framework for transferring research to practice. J Subst Abus Treat.

[41] **Azevedo KJ, Gray CP, Gale RC, et al**. Facilitators and barriers to the Lean Enterprise Transformation program at the Veterans Health Administration. Health Care Manag Rev*.* 2020. 10.1097/HMR.0000000000000270 31996609

[42] Peden CJ, Stephens T, Martin G (2019). Effectiveness of a national quality improvement programme to improve survival after emergency abdominal surgery (EPOCH): a stepped-wedge cluster-randomised trial. Lancet.

[43] Perla RJ, Bradbury E, Gunther-Murphy C (2013). Large-scale improvement initiatives in healthcare: a scan of the literature. J Healthc Qual.

[44] Boushon B, Provost L, Gagnon J, Carver P (2006). Using a virtual breakthrough series collaborative to improve access in primary care. Jt Comm J Qual Patient Saf.

[45] Butler A, Canamucio A, Macpherson D, Skoko J, True G (2014). Primary care staff perspectives on a virtual learning collaborative to support medical home implementation. J Gen Intern Med.

[46] Speroff T, Ely E, Greevy R (2011). Quality improvement projects targeting health care–associated infections: comparing virtual collaborative and toolkit approaches. J Hosp Med.

[47] Zubkoff L, Neily J, King BJ (2016). Virtual breakthrough series, part 1: preventing catheter-associated urinary tract infection and hospital-acquired pressure ulcers in the veterans health administration. Jt Comm J Qual Patient Saf.

[48] Zubkoff L, Neily J, Quigley P (2016). Virtual breakthrough series, part 2: improving fall prevention practices in the veterans health administration. Jt Comm J Qual Patient Saf.

[49] Zubkoff L, Neily J, King B (2017). Preventing pressure ulcers in the Veterans Health Administration using a virtual breakthrough series collaborative. J Nurs Care Qual.

[50] Watts B, Norton WE (2016). Learning from the virtual breakthrough series collaboratives in the Veterans Health Administration. Jt Comm J Qual Patient Saf.

[51] **Rotenstein LS, Johnson AK.** Taking back control—can quality improvement enhance the physician experience? In. Health Affairs Blog; 2020. 10.1377/hblog20200110.543513

[52] Swensen SJ, Dilling JA, Mc Carty PM, Bolton JW, Harper CM (2013). The business case for health-care quality improvement. J Patient Saf.

[53] Baron AN, Hemler JR, Sweeney SM (2020). Effects of practice turnover on primary care quality improvement implementation. Am J Med Qual.

[54] Strating MM, Nieboer AP (2013). Explaining variation in perceived team effectiveness: results from eleven quality improvement collaboratives. J Clin Nurs.

[55] Bradley EH, Brewster AL, McNatt Z (2018). How guiding coalitions promote positive culture change in hospitals: a longitudinal mixed methods interventional study. BMJ Qual Saf.

[56] McCarthy D, Blumenthal D (2006). Stories from the sharp end: case studies in safety improvement. Milbank Q.

[57] Babich LP, Charns MP, McIntosh N (2016). Building systemwide improvement capability: does an organization’s strategy for quality improvement matter?. Qual Manag Health Care.

[58] Cohen DJ, Balasubramanian BA, Gordon L (2016). A national evaluation of a dissemination and implementation initiative to enhance primary care practice capacity and improve cardiovascular disease care: the ESCALATES study protocol. Implement Sci.

[59] VanDeusen Lukas CV, Holmes SK, Cohen AB (2007). Transformational change in health care systems: an organizational model. Health Care Manag Rev.

