# Supplementary material for: The LEAP Program: Quality Improvement Training to Address Team Readiness Gaps Identified by Implementation Science Findings
Source: J Gen Intern Med. 2020 Sep 8;36(2):288–95. doi: 10.1007/s11606-020-06133-1 (PMC7878618; doi:10.1007/s11606-020-06133-1)
Supplement: Supplementary file 1 — (DOCX 251 kb). [file 11606_2020_6133_MOESM1_ESM.docx]

# **Appendix 1: Project Charter Template**

# **Project Charter**

**Name:**

**Team Members (if applicable):**

**Project Title:**

***What are we trying to accomplish?***

Problem to be addressed (2-3 sentences)

- Broadly speaking, *WHAT* is the problem you want to address?
- It could be a problem with a process (e.g., patients are having to wait 6 weeks to start MOVE!) or a patient outcome (e.g., 60% of patients in MOVE! do not lose any weight)
- Be specific and compelling

Reason for the effort (4-5 sentences)

- *WHY* is it important to address the problem? (link to VA/VAMC goals, priorities, or strategies)
- Consider why your colleagues or leaders should care about addressing this problem – what would you say to motivate them to support/help you address the problem?

Expected outcomes/benefits (3-4 sentences)

- If the problem were addressed, WHAT specific outcomes would you expect? (but still not *HOW*)

**AIM STATEMENT** (1-2 sentences)

- A clear statement that describes *what* is to be improved, by *how much* (numeric goal), *by when* (timeline), and for *whom* (who will benefit)
- With the support of… (a sponsor or leader who helps overcome barriers, resource challenges, etc.)

- Be sure to include the boundaries of your efforts. E.g., with the Blue and Purple primary care teams

***How do we know that a change is an improvement?*** (4-5 sentences)

- Identify outcome, process, and balancing measures that can be collected at intervals frequent enough to assess progress on the project; 4-5 sentences)

*Outcome measure(s):*

*Process measure(s):*

*Balancing measure(s):*

***What are the barriers to success?***

- Using the feedback from tools like the Barrier Buster Tool and Fishbone Diagram, list the specific barriers that your team will need to address to make the improvement listed in your Project Aim.

***What changes can we make that will lead to improvement?*** (4-5 sentences)

- List 1-3 ideas of small, feasible changes and state how or why you think they will help you achieve your aim.

[Change X] will result in [outcome Y] because [reason Z].

***What are the constraints within which we must accomplish the change?*** (2-3 sentences)

- These are constraints on the scope of the project (e.g., parameters around target population, project duration, time, money, resources, size of project, or support from leadership).
- What will your project *not* address but if successful, you might address or expand in the next PDSA cycle?

**Appendix 2:**

**Description of the Learn. Engage. Act. Process. (LEAP) Program**


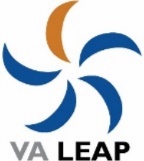
**LEAP Design Goals**

The Learn. Engage. Act. Process. (LEAP) program is designed to engage frontline clinical teams in quality improvement (QI) using a hands-on learning approach. By the end of LEAP, teams will complete a QI project with the help of a coach and with support from a learning community comprised of other teams. Team members come away from LEAP with higher confidence in applying QI methods to improve quality of care within the demands of everyday clinical practice and intentions to continue QI to optimize care for their patients.

LEAP design was driven by the belief that all health system employees have the power to make healthcare better for their patients, even within the reality of busy clinics with competing priorities that too often lead to limited time and resources. We designed LEAP specifically for these busy frontline teams and coach them through weekly participation in a team-developed improvement project. LEAP is virtually delivered, requiring no travel. Learning is accomplished in a paced fashion that minimizes cognitive load and is sensitive to the time commitment. Teams are coached in “cohorts” of multiple teams that have the opportunity to build a lasting supportive learning community. Materials and curriculum are based on the Institute for Healthcare Improvement’s (IHI) Model for Improvement, which uses plain, everyday language that minimizes technical jargon. Teams choose their aims and develop projects based on their priorities within a broad topic, such as improving obesity treatment or increasing patient access to services by reducing unnecessary endoscopy procedures.

**LEAP Helps Achieve Team and Health System Goals**

A LEAP Coach works with local teams to accomplish the following:

| 1. Build and engage an improvement team | 6. Outline specific changes to test |
| --- | --- |
| 1. Brainstorm opportunities for improvement | 7. Formulate a data collection plan and monitor changes |
| 1. Explore root causes for areas for improvement | 8. Reflect and refine project changes/measures |
| 1. Develop an aim | 9. Communicate project progress |
| 1. Create a project charter | 10. Plan for future cycles of change to sustain, scale up, and spread improvements |

With health system leaders and operational partners, we identify broad topics with which frontline teams may engage. For example, in our “LEAP for MOVE!” (VA’s weight management program) project, national leaders wanted to reduce the wide variation in how group-based MOVE! treatment was structured and delivered at the approximately 150 medical centers within VA. On other projects, we are working with system leaders to activate frontline teams to achieve goals related to building a culture of continuous process improvement, reducing unnecessary endoscopy procedures, and improving rates of appropriate medication use. These are all examples of broad topics within which local teams develop their own projects based on their local priorities. We recognize that the diversity of settings, even within a single health system, means diversity in local workflows and in priorities for improvement; individuals working at the frontline are the experts in how things work within their own clinics and thus, experts in how best to optimize and improve patient care.

**Engaging Frontline Teams in LEAP**

Within each topic area, we work with local facility partners to find a willing and interested volunteer; the most effective leaders are individuals who have intrinsic motivation and commitment to improving care within a topic. In our MOVE! project, this individual was usually the MOVE! Program Coordinator who took on the role of LEAP Team Leader, the person who leads the local improvement team through a QI project from beginning to end with the support of a LEAP Coach. Prior to starting LEAP, our staff works with local facility staff to facilitate access to needed program metrics, to test webinar technical equipment, and to agree to a start date. Figure 1 provides an overview of the LEAP curriculum. Our staff work with the Team Leader to schedule weekly sessions in a way that works well with their clinical commitments.

**Figure 1. LEAP Curriculum At-A-Glance.**


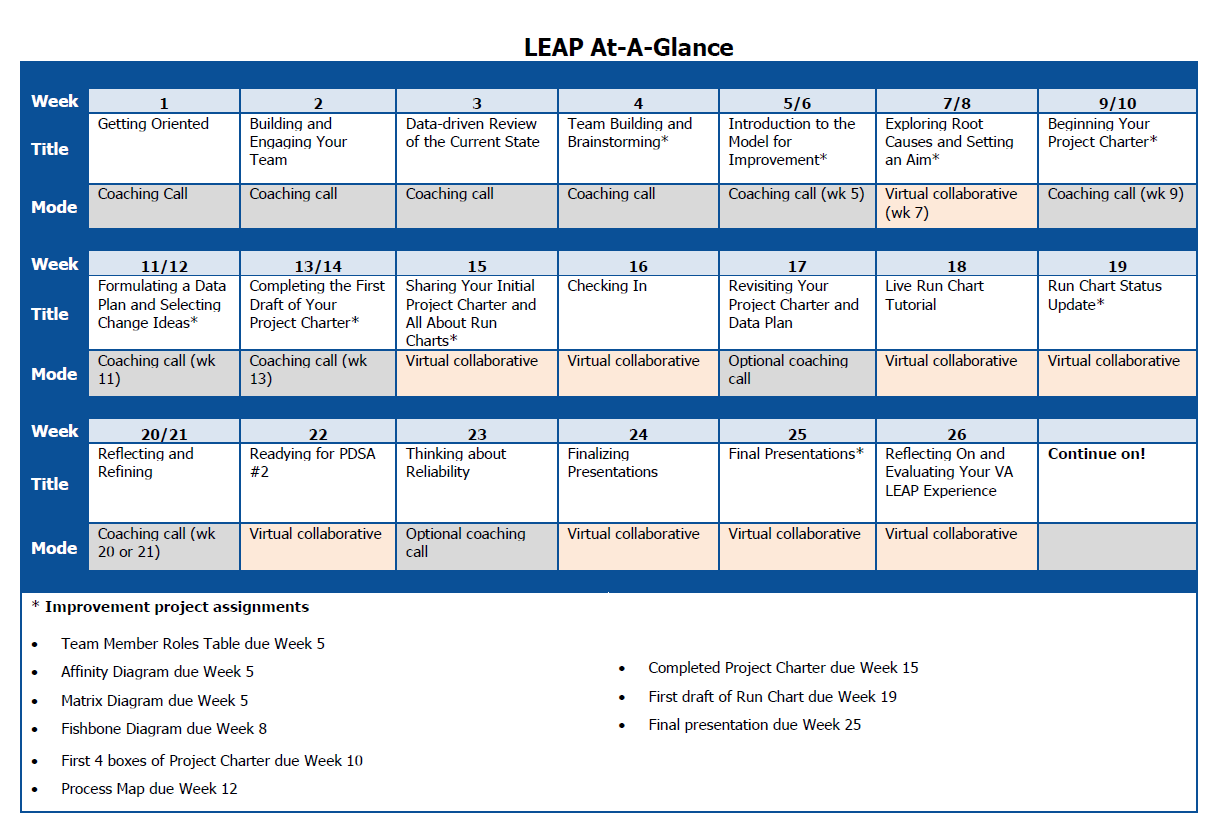


In the first four weeks, the LEAP coach works with the Team Leader (and existing team members if pre-identified and already committed) to form a robust interdisciplinary LEAP team. Written guidance emphasizes the importance of assembling a team that has a shared commitment and sense of purpose. Recommended team size is 5-8 members who represent a diversity of roles and perspectives. LEAP teams are encouraged to include a patient because they are the ultimate recipient of care. Effective teams include members representing three different kinds of expertise within the organization: leaders/managers, subject-area experts, and co-workers. Teams are encouraged to clarify and define the role of each member.

**LEAP Process and Time Allocation**

**Figure 2:** Example of Weekly Overview and Task Checklist


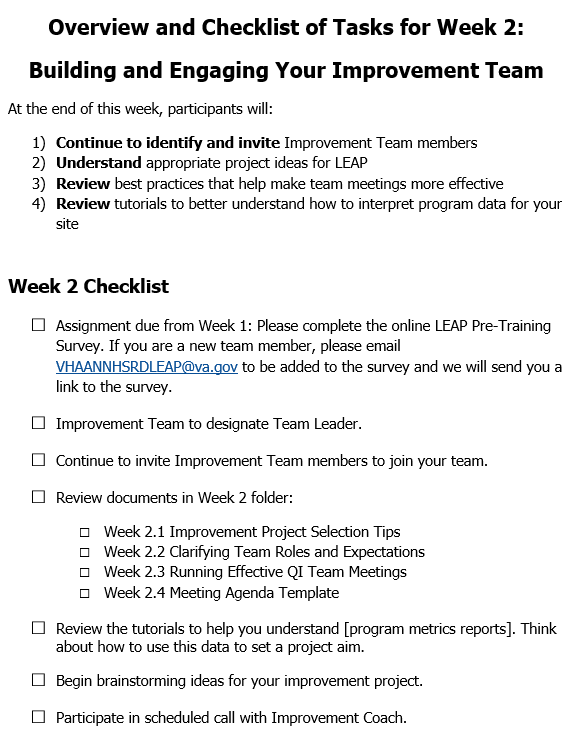
Every Friday, teams are emailed a checklist for the coming week (Figure 2). The Team Leader expends the most hours while participating in LEAP, while other team members vary in their time commitment based on their team role. Most weeks, the Team Leader will spend an hour with the LEAP coach in a webinar format either alone or with other LEAP team members. Early in the program, these sessions are focused on individual teams, but later in the program, sessions are increasingly collaborative, including Team Leaders plus members from other teams in a cohort of up to six teams as shown in Figure 1 (e.g., the first five weeks, the coach works with individual teams and then in Week 7, the coach facilitates the first collaborative session with all LEAP teams in the cohort). We have also built in “working weeks,” during which there is not a scheduled coaching call or virtual collaborative session, allowing time and space for the local team to meet and accomplish project-related tasks.

Virtual collaborative sessions are designed to provide teams time and space to connect with their peers. LEAP coaches deliver limited didactic content during the collaboratives because it is primarily meant to be an open forum for teams to share their barriers and facilitators to progress, help problem-solve, and to build a supportive learning community. The goal is to build connections between teams with less reliance on the LEAP coach. All teams work on the same topic (e.g., improving obesity treatment), but develop their own specific projects with a variety of specific aims.

In addition to the weekly coaching sessions (individual or collaborative sessions), the Team Leader schedules local team meetings to work on the week’s assignments. For example, in Week 2 (see Figure 2), local teams meet to discuss roles, membership, and start to brainstorm improvement ideas. The Team Leader will also expend hours in tasks necessary to accomplish their chosen project, like identifying and recruiting team members and coordinating with a supervisor, and ultimately, takes on the primary role of executing the planned change, monitoring progress, and problem-solving. Typically, Team Leaders can expect to dedicate 3-5 hours per week; team members often expend much less time ranging from 1 to 5 hours, depending on the week (stage of the project) and their role.

**Online Platform**

LEAP has an online platform where all resources (written documents, templates, tools, videos) are available for teams. Teams post their assignments (e.g., Project Charter) online to share with other teams within their cohort and with their LEAP Coach. There is also a discussion area and when feasible, a portal to data reports that help inform QI projects. This platform was hosted with VA Pulse for early cohorts of teams, which is a social-networking platform, however the LEAP platform is being migrated to Microsoft’s SharePoint Online.

**Source of LEAP Curriculum**

The LEAP curriculum is paced to fit a frontline employee’s busy clinical schedule. Curriculum was adapted from a Massive-Open Online Course (MOOC) developed by HarvardX in collaboration with IHI.^1^ Materials from the MOOC were adapted for LEAP by: 1) designing for teams rather than individuals, 2) streamlining materials to accommodate busy front-line team members, and 3) lengthening program duration to provide more time to complete a project.

**LEAP Coaches and Supervision**

LEAP Coaches have a college degree (most are masters-level) in a health-related field (e.g., social work, public health) with experience working in a healthcare setting. All coaches are trained by an experienced LEAP Coach supervisor and complete the IHI Improvement Coach Professional Development Program or equivalent. All coaching sessions are audio recorded. Recordings are used to guide weekly peer supervision and fidelity sessions, attended by all LEAP coaches.

**Sample Team Experience**

We provide a high-level description of one team’s experience with LEAP, focused on improving MOVE! obesity treatment. Their long-term goal was to incorporate patient-centered care concepts into their program using an approach that empowers and equips patients to take charge of their health in a personally meaningful way.^2^ These concepts are aligned with evidence-based interventions for achieving healthy weights. Their overall goal was to “explicitly identify a way for Veterans to connect their weight loss goals to their values and life aspirations that will enhance motivation and treatment outcomes; to generate a discussion that allows them to explore the drive behind wanting to lose weight.” They worked with their LEAP coach in the first months of the program to articulate the reason for their goal, expected outcomes, and a specific aim:

*We aim for 90% of Veterans enrolled in 16-week MOVE program to complete their Whole Health Score Card on session 1. We will test this change with the next new 16-week MOVE cohort that starts [date] through the first 10 weeks of the program…*

They identified outcome measures they wanted to impact (e.g., average pounds of weight lost in 10 weeks) and developed a specific improvement (development of a scorecard for their patients), along with process measures (ensuring each patient received their scorecard) and their theory about why they believed giving scorecards would help patients lose more weight (the scorecard will help patients self-monitor, which is a key to making behavior changes leading to weight loss). They also identified potential barriers and mitigation strategies. They worked with their LEAP coach to develop a run chart to track progress with scorecard implementation. In the last weeks of LEAP, they presented their results and experiences in a collaborative session with other teams in their cohort. They provided lessons learned and plans for future cycles of change. This team has continued to work on improvement projects as described in follow-up interviews. Team members described how their LEAP experience and subsequent quality improvement work has increased collegiality saying, “We’ve gone beyond our silos.”

Other teams have affirmed this experience saying e.g., that “[LEAP] really sparked the most conversation and discussion about the current process, identifying opportunities for improvement…we really thought through the whole reason of, why are we doing this…and identified specific measures we were striving for…” and, “Expectations for each week and a check list has made it very manageable.”

**Summary**

LEAP offers frontline employees virtual QI training that is supportive, user-friendly, and immediately relevant. LEAP helps frontline teams develop projects that address priorities within their local setting and mitigate barriers to implementing changes by fostering skills in QI methods, open communications, developing productive working relationships, and building team-based problem-solving skills.

Gaps in team-level capability to accomplish implementation goals or participate in QI are well-documented within and outside VA. The imperative for everyday QI is reinforced by the Dynamic Sustainability Framework, which insists that a dynamic, incremental process of adaptations is necessary to optimize fit between new programs and sustain use over the long-term. Everyday QI is essential for high-quality patient care, especially in a constantly changing landscape of medical breakthroughs that need to be reliably implemented. More and more health systems aim to become learning health systems and/or High Reliability Organizations (HRO). Clinical teams engaged in QI as a part of everyday work practices is at the heart of both of these laudable goals. Engaging teams in continuous QI is enormously challenging, and yet, doing so has potential for many positive collateral effects as well including giving clinicians and staff a strong sense of ownership in their work, bringing meaning and purpose to work-life, and reducing rising rates of burnout.

**References**

1. EdX, Inc. Practical Improvement Science in Health Care: A Roadmap for Getting Results. <https://www.edx.org/course/ph556x-practical-improvement-science-in-health-care-a-roadmap-for-getting-results>. Accessed January 15, 2019.

2. Hill JN, Locatelli SM, Bokhour BG, et al. Evaluating broad-scale system change using the Consolidated Framework for Implementation Research: challenges and strategies to overcome them. *BMC Res Notes.* 2018;11(1):560.

**Appendix 3: Organizational Readiness for Implementing Change Assessment**

**Page 1 - Week 5 (Pre-LEAP ORIC):**

This survey has 12 questions to help the LEAP staff understand the environment within which you are attempting to enact changes for MOVE!. You may not yet have decided on your change. That’s ok; imagine you do have a change in mind and provide answers based on that.

Please have each team member on your Improvement Team fill out this survey. An identical survey will be administered at the end of the LEAP program.

**Page 1 - Week 20 (Post-LEAP ORIC):**

You may recall taking this survey in Week 5 of LEAP. As a reminder, there are 12 questions to help the LEAP staff understand the environment within which you are attempting to enact changes for MOVE!. For this survey, please consider a ***future*** change to MOVE! when responding to each item.

Please have each team member on your Improvement Team fill out this survey. We will report your scores from Week 5 and from this survey back to you so you can compare how your environment may have changed.

**Page 2**

Each item asks about “people”. You may include yourself, anyone on your LEAP team, and others who may support or participate with you to implement the planned change.

As you consider your planned change to MOVE!, please indicate your degree of agreement or disagreement with each of the following statements.

| 1 | 2 | 3 | 4 | 5 |
| --- | --- | --- | --- | --- |
| Disagree | Somewhat  Disagree | Neither Agree nor Disagree | Somewhat  Agree | Agree |

| 1. People who work here feel confident that the organization can get people invested in implementing this change. |
| --- |
| 1. People who work here are committed to implementing this change. |
| 1. People who work here feel confident that they can keep track of progress in implementing this change. |
| 1. People who work here will do whatever it takes to implement this change. |
| 1. People who work here feel confident that the organization can support people as they adjust to this change. |
| 1. People who work here want to implement this change. |
| 1. People who work here feel confident that they can keep the momentum going in implementing this change. |
| 1. People who work here feel confident that they can handle the challenges that might arise in implementing this change. |
| 1. People who work here are determined to implement this change. |
| 1. People who work here feel confident that they can coordinate tasks so that implementation goes smoothly. |
| 1. People who work here are motivated to implement this change. |
| 1. People who work here feel confident that they can manage the politics of implementing this change. |

**Page 3**

To allow the LEAP staff to provide you with the survey data for your site, please enter your VA facility name below.

(free text response – mandatory response)

To allow the LEAP team to link your Pre-LEAP ORIC assessment with the Post-LEAP ORIC assessment you will take at the end of the LEAP program, please enter your email address below

(free text response – mandatory response)

**Scoring: Compute the following scores:**

Collective confidence in making this change

- Average of 1, 3, 5, 7, 8, 10,12

Collective commitment to making this change

- Average of 2, 4, 6, 9, 11

Overall readiness to make this change

- Average of all items

Calculate statistically significant change (p<.05), using paired t-tests, if possible

**Appendix 4: Quality Improvement Skills Self-Assessment Survey**

**Page 1 - Pre-LEAP Self-Assessment (introduction)**

This self-assessment will help you assess your quality improvement skills during your participation in LEAP. There will be identical surveys administered at the beginning and end of the LEAP program. 
Thank you in advance for taking the time to complete this self-assessment

**Page 1 - Post-LEAP Self-Assessment (introduction)**

This post-assessment will help you assess your quality improvement skills during your participation in LEAP. This survey is identical to the assessment survey administered at the beginning of the LEAP program.

Please have **each team member** on your Improvement Team fill out this survey.
Thank you in advance for taking the time to complete this self-assessment.

**Page 1 – Both surveys**

This self-assessment has 19 skills that are divided into six skill areas. For each skill area, select the one response that best describes your skill level. There is no wrong answer and this is not a test. We will provide a report back to you that compares these responses to your earlier responses.

The response options are:

**(1) No Knowledge:** “I cannot tell you what this skill, tool, or method is.”
**(2) Knowledge:** “I can tell you what this skill, tool, or method is and give you facts about it.”
**(3) Basic Application:** “I can tell you what this skill, tool, or method is and given a defined situation, I can apply it with assistance.”
**(4) Analysis and Application:** “I have knowledge of the skill, tool, or method and I can analyze a situation and determine if it is needed, and then independently and accurately apply it.”
**(5) Highly Experienced:** “I have knowledge of this skill, tool, or method, I have a high degree of experience correctly applying and adapting it in various situations, and I can explain my decisions for doing so.”
**(6) Expert:** “I have knowledge of this skill, tool, or method, I have a high degree of experience correctly applying and adapting it, and I can teach others the theory behind it and coach them in its use.”

**Page 2 -** (Response options repeated at top of page)

**Skill Area 1: Support a Change with Data**

1. Develop a family of measures (i.e. process, outcome, and balancing measures)

2. Build clear and unambiguous operation definitions for measures

3. Develop practical data collection plans

4. Construct and interpret a run chart

**Page 3 -** (Response options repeated at top of page)

**Skill Area 2: Develop a Change**

5. Decide how large a change is actually needed (e.g. variation in a process versus create a whole new process)

6. Use flowcharts, cause/effect diagrams, driver diagrams or other useful tools to document the system to be changed

7. Use flowcharting techniques to break a system down into numerous sub-processes that describe how work gets done

8. Identify specific ideas that will achieve the desired results

**Page 4 -** (Response options repeated at top of page)

**Skill Area 3: Testing a Change**

9. Design, set up, and run Plan-Do-Study-Act cycles

10. Run tests on a small scale initially and then increase the scale and scope of testing as you learn

11. Develop qualitative and/or quantitative data collection plans for the Plan-Do-Study-Act cycle

**Page 5 -** (Response options repeated at top of page)

**Skill Area 4: Implementing a Change**

12. Develop new structures and procedures to support the implemented change (e.g. training, new policies and procedures, new equipment)

13. Create measurement approaches to determine if the improvements observed during the testing stage have been sustained during implementation

**Page 6 -** (Response options repeated at top of page)

**Skill Area 5: Spreading a Change**

14. Distinguish clearly how testing, implementing, and spreading a change are all different steps in the sequence of improvement

15. Create communication strategies that support spread

**Page 7 -** (Response options repeated at top of page)

**Skill Area 6: The Human Side of Change**

16. Plan and conduct effective team meetings (e.g. set agendas assign roles such as recorder and time keeper, establishing ground rules for behavior)

17. Use decision making tools such as Nominal Group Technique, Multi-Vote, Rank Order and Structured Discussion to arrive at team consensus for decisions

18. Model active listening for others

19. Handle difficult conversations when individuals express opposing views

**Appendix 5: LEAP Program Satisfaction Survey**

**Page 1**

Q1. Thank you for participating in the LEAP program!

In an effort to improve LEAP for future participants, we would like to know more about your experiences and satisfaction with LEAP and what you think we can do to improve the program.

We would be very grateful if **each Improvement Team member** at your facility would take a few minutes to complete the following survey, which should take 10 minutes or less to complete.

Q2. What is your VA facility?

Q3. What is your role in the LEAP Improvement Team at your VA facility?

- Improvement Team Leader
- Improvement Team member (not the leader)
- Improvement Team supporter
- Other (please describe)

Q4. If you selected Other above, please describe your role here. (free text response)

**Page 2**

Q5. Please indicate your response to the following statements about your experience in LEAP, using the following scale.

| 1 | 2 | 3 | 4 | 5 |
| --- | --- | --- | --- | --- |
| Strongly Disagree | Disagree | Neither Disagree nor Agree | Agree | Strongly Agree |

| The LEAP program is relevant to the needs of our MOVE! program. |
| --- |
| I feel comfortable using the LEAP materials and methods to help guide improvements to our MOVE! program. |
| I had the time to do the work required during the 21-week LEAP program. |
| Our program had sufficient resources to implement program improvement methods learned during LEAP. |

Q6. If you responded with a 3 or below for any of the above items, what could be done to improve your experience with those components of LEAP? (free text response)

**Page 3**

Q7. Please indicate your response to the following statements about your intentions after LEAP, using the following scale.

| 1 | 2 | 3 | 4 | 5 |
| --- | --- | --- | --- | --- |
| Strongly Disagree | Disagree | Neither Disagree nor Agree | Agree | Strongly Agree |

| Our LEAP Improvement Team will continue working together after the 21 weeks of LEAP. |
| --- |
| I will have the time to continue to apply LEAP methods to improve our MOVE! program in the future. |
| Follow-up support (coaching or virtual collaborative sessions) will help me use what I learned through LEAP to continue improving our MOVE! program. |
| I plan to attend follow-up coaching or virtual collaborative sessions. |
| I plan to invite other staff from our facility to attend follow-up coaching or virtual collaborative sessions. |
| I plan to continue to monitor our MOVE! program using the MOVE! data reports provided by LEAP. |

Q8. How could the LEAP staff help support you to continue to apply LEAP methods to improve your MOVE! program in the future? (free text response)

**Page 4**

Q9. Please indicate your level of satisfaction with the components of LEAP below, using the following scale.

| 1 | 2 | 3 | 4 | 5 |
| --- | --- | --- | --- | --- |
| Very Unsatisfied | Unsatisfied | Neither Unsatisfied nor Satisfied | Satisfied | Very Satisfied |

| Support provided by the Improvement Coaches |
| --- |
| Sources of the LEAP material (e.g., IHI, Harvard X, The Improvement Guide) |
| Quality of the written material |
| Quality of the videos |
| Organization of the LEAP material on VA Pulse |
| Navigation on the VA Pulse LEAP groups |
| Number of assignments |
| Technology requirements of the LEAP program |

Q10. If you responded with a 3 or below for any of the above items, what could be done to improve your level of satisfaction with those components of LEAP? (free text response)
